# Universal health coverage from multiple perspectives: a synthesis of conceptual literature and global debates

**DOI:** 10.1186/s12914-015-0056-9

**Published:** 2015-07-04

**Authors:** Gilbert Abotisem Abiiro, Manuela De Allegri

**Affiliations:** Institute of Public Health, Medical Faculty, University of Heidelberg, Heidelberg, Germany; Department of Planning and Management, Faculty of Planning and Land Management, University for Development Studies, University Post Box 3, Wa, Upper West Region Ghana

**Keywords:** Universal health coverage, Multi-dimensional concept, Rights-based, Population coverage, Financial protection, Access to health services, Health system, Conceptual literature, Global debates

## Abstract

**Background:**

There is an emerging global consensus on the importance of universal health coverage (UHC), but no unanimity on the conceptual definition and scope of UHC, whether UHC is achievable or not, how to move towards it, common indicators for measuring its progress, and its long-term sustainability. This has resulted in various interpretations of the concept, emanating from different disciplinary perspectives. This paper discusses the various dimensions of UHC emerging from these interpretations and argues for the need to pay attention to the complex interactions across the various components of a health system in the pursuit of UHC as a legal human rights issue.

**Discussion:**

The literature presents UHC as a multi-dimensional concept, operationalized in terms of universal population coverage, universal financial protection, and universal access to quality health care, anchored on the basis of health care as an international legal obligation grounded in international human rights laws. As a legal concept, UHC implies the existence of a legal framework that mandates national governments to provide health care to all residents while compelling the international community to support poor nations in implementing this right. As a humanitarian social concept, UHC aims at achieving universal population coverage by enrolling all residents into health-related social security systems and securing equitable entitlements to the benefits from the health system for all. As a health economics concept, UHC guarantees financial protection by providing a shield against the catastrophic and impoverishing consequences of out-of-pocket expenditure, through the implementation of pooled prepaid financing systems. As a public health concept, UHC has attracted several controversies regarding which services should be covered: comprehensive services vs. minimum basic package, and priority disease-specific interventions vs. primary health care.

**Summary:**

As a multi-dimensional concept, grounded in international human rights laws, the move towards UHC in LMICs requires all states to effectively recognize the right to health in their national constitutions. It also requires a human rights-focused integrated approach to health service delivery that recognizes the health system as a complex phenomenon with interlinked functional units whose effective interaction are essential to reach the equilibrium called UHC.

## Background

Universal health coverage (UHC) has been acknowledged as a priority goal of every health system [1–5]. The importance of this goal is reflected in the consistent calls by the World Health Organization (WHO) for its member states to implement pooled prepaid health care financing systems that promote access to quality health care and provide households with the needed protection from the catastrophic consequences of out-of-pocket (OOP) health-related payments [2, 6–8]. This call has also been endorsed by the United Nations [5].

In the existing literature, different conceptual terminology, such as universal health care [9], universal health care coverage [10, 11], universal health system, universal health coverage, or simply universal coverage, have been used to refer to basically the same concept [9, 12–14]. Stuckler et al. [15] noted that “universal health care” is often used to describe health care reforms in high income countries while “universal health coverage” is associated with health system reforms within low- and middle-income countries (LMICs). Given that the poor, marginalized and most vulnerable populations mostly reside in LMICs, this paper places relatively high emphasis on such settings. Hence, we adopt the term universal health coverage (UHC) [2] throughout the paper.

It is argued that health system reforms aimed at UHC can be traced back to the emergence of organized health care in the 19th century, in response to labor agitations calling for the implementation of social security systems [16–18]. This phenomenon first started in Germany under the leadership of Otto von Bismarck, and later spread throughout other parts of Europe such as Britain, France and Sweden [16–18]. Later in 1948, the concept of UHC was implicitly enshrined in the WHO constitution which recognized that *“the enjoyment of the highest attainable standard of health is one of the fundamental rights of every human being without distinction of race, religion, and political belief, economic or social condition*” [19]. This fundamental human right was reaffirmed in the “*Health for all*” declaration of the Alma Ata conference on primary health care in 1978 [20].

In 2005, the concept of UHC was once again acknowledged and for the first time explicitly endorsed by the World Health Assembly (WHA) as the goal of sustainable health care financing [6]. The World Health Assembly resolution (WHA58.33) explicitly called for the implementation of health care financing systems centered on prepaid and pooling mechanisms aimed at achieving UHC [6]. Based on this Resolution, WHO defined UHC as *“access to key promotive, preventive, curative and rehabilitative health interventions for all at an affordable cost, thereby achieving equity in access”* [6]*.* The 2008 World Health Report re-emphasized prepayment and pooling systems as essential instruments for UHC by categorically stating that UHC entails “*pooling pre-paid contributions collected on the basis of ability to pay, and using these funds to ensure that services are available, accessible and produce quality care for those who need them, without exposing them to the risk of catastrophic expenditures”* [7]*.* In 2010, the World Health Report, further stressed the role of health system financing for UHC by arguing that “*countries must raise sufficient funds, reduce the reliance on direct payments to finance services, and improve efficiency and equity”* [2]*.* The concept of UHC as reflected in these WHO reports seems to be focused more on improving the health care financing function of a health system. The 2013 World Health Report built on prior work resulting in a call for research evidence to facilitate the transition of countries towards UHC [8]. The United Nations, the World Bank, the Gates Foundation, Oxfam, United States Agency for International Development (USAID), the International Labour Organization, United Nations Children’s Fund (UNICEF), Rockefeller Foundation, Results for Development Institute, the Joint Learning Network, among other international and regional development organizations have also in various ways recently endorsed and promoted the move towards UHC [5, 21–25]. Considering the key role of the WHO and these other global actors in shaping the health policy debate at the global level, this recent history demonstrates a consistent and increasing international interest in the concept and debates surrounding UHC [2, 26].

To date, the literature continues to present a clear consensus on the importance of UHC [23, 27–29]. UHC was described by the Director General of WHO as “*the single most powerful concept that public health has to offer”* [30]. Its potential to improve the health of the population, especially for the poor, has been demonstrated [31, 32]. It is viewed as the phenomenon that will result in the third global transition and hence greatly influence the (re-) organization and financing of global health systems [29]. As an essential catalyst for poverty reduction and economic growth [14, 33, 34], UHC is regarded as a prerequisite for sustainable development [35]. It has therefore been advocated for as an important health goal in the post-2015 global development agenda [35–40]. The Lancet Commission on Investing in Health reports that this goal can be progressively attained by 2035 [34].

Despite the global consensus on its importance, consensus on the conceptual definition, meaning, and scope of UHC are still missing [12, 26, 41]. Likewise, no consensus exists on whether UHC is achievable or not; on how to move towards it [3, 22, 42, 43]; on common indicators for measuring progress towards it [13, 24, 28, 29, 44]; and on its long-term sustainability [27]. The absence of a clear consensus on the conceptual definition of UHC has resulted in various interpretations of the concept, emanating from different disciplinary perspectives. These different interpretations reveal distinct, but interlinked dimensions of UHC [2]. This paper seeks to explore these various interpretations and representations of the concept of UHC from a multidimensional perspective and to discuss the various dimensions of UHC emerging from these interpretations. The arguments presented in this paper are based on a synthesis of the literature emerging from recent global debates on UHC. We adapted the WHO framework [2] to guide the presentation of our synthesis of the conceptual debates currently being advanced in the literature. Inspired by the WHO framework, our conceptual reasoning is that advancing UHC requires a healthy interaction across the three coverage dimensions: population coverage, financial protection and access to health services, held together by the view of health as a legal human right.

## Discussions

### Universal health coverage as a legal right to health

A group of scholars, building their opinions from a legal and human rights perspective, enshrined in various international covenants and treaties [45–49], argue that the concept of UHC implies the existence of a legal framework to ensure that every resident gets access to affordable health care [15, 50, 51]. This portrays UHC as a reformulation of the “*health for all”* goal of the Alma Ata Declaration [15, 22, 52–54]. The view of UHC as a legal obligation imposed on all states that ratified the convention on the right to health [45], implies that UHC calls for all States to create legal entitlements to health care for all their residents [50, 55, 56], thereby placing the responsibility for the delivery of UHC on national governments [5, 17, 57]. To guarantee a comprehensive right to health, the legal obligation of the state needs to reach beyond mere health service provision to include deliberate efforts to advance improvements in structures which are recognized to act as important social determinants of health such as, education, housing, sanitation and portable water as well as equitable gender and power relations [58–60]. The goal of UHC and the responsibility of moving towards it, therefore, need to be mandated by national laws [4, 61, 62]. Backman et al. [63] report that only 56 states have constitutional provisions that legally recognize the right to health and argue that even within these states, much work is still needed to ensure that this right is guaranteed in actual practice for all. Kingston et al. [55] also argue that even the state-centered view of the right to health is based on a false assumption that all people have legal nationalities. They insist that this false assumption is the cause for the medical exclusion of some migrants, especially illegal immigrants, from accessing institutionalized health care within their countries of residence. This situation is even more serious in LIMCs, where states find it difficult to raise sufficient revenues to finance health care for their legal citizenry. The vague definition of the right to health for non-nationals premised on the individual state’s economic ability and willingness to guarantee it [46], is therefore a potential recipe for social exclusion on the basis of nationality. Current debates on UHC therefore need to seriously reflect on ways by which the rights of stateless individuals to health care can also be guaranteed within the framework of UHC.

Acknowledging financial constraints to enforcing the right to health within poor-resource settings, some scholars explicitly call for international assistance for health as a way of strengthening the right to health component of UHC [62, 64]. This, they argue, can be implemented through the establishment of a global fund to finance UHC [65] thereby presenting health as a global public good [66]. The notion of creating a common fund for UHC also recognizes the transnational nature of emerging global health problems and the inherent global interdependency needed to deal with such problems [67]. The possibility of funding global efforts towards UHC from this global fund is being explored. Initial results reveal conflicting expectations and interests between the potential donors/financiers and beneficiary countries [65]. The rights-based arguments for UHC therefore suggest a shift on the ethical spectrum of international assistance for health, from the concept of international health, where international assistance for health is viewed as a form of charity, towards that of global health [62, 67–69] which is driven by the cosmopolitan ethical preposition that states should assist each other on the basis of humanitarian responsibility [68, 69] and solidarity [67]. This cosmopolitan ethical view has the potential of facilitating efforts at raising more international assistance to facilitate UHC within its broader dimensions currently being advanced by WHO and other global experts.

### Population coverage as a dimension of universal health coverage

Another group of scholars [22, 61], also supportive of the rights-based perspective, argue that UHC implies “*equal or same entitlements”* to the benefits of a health system. This reflects the notion of universal enrollment into health-related social security or risk protection systems [17, 70] or population coverage under public health financing systems [2]. This notion therefore puts people (population) at the center of UHC [71]. Universal population coverage is to be understood in relation to the tenets of the right to health [45] as the absence of systemic exclusion of certain population groups (especially the poor and vulnerable) from the coverage of public prepaid funds and the ability of all residents to enjoy the same entitlements to the benefits of such public funding, irrespective of their nationality, race, sexual orientation, gender, political affiliations, socio-economic status or geographic locations [2, 12, 22, 53, 55, 61, 72–74].

To distinguish between aggregate and equity-based measures of population coverage, both WHO & the World Bank [24] have defined population coverage along two dimensions. Thus; achieving a 100 % coverage of the total population as an aggregate measure, or ensuring a relatively good proportion of coverage of the poorest 40 % compared to the rest of the population as an equity-based measure [24]. The overall notion of equity, defined as progressive income-rated contributions to health financing and need-based entitlements to health services, is embedded in almost all conceptual definitions of universal population coverage [2, 4, 75–77]. Implicit in the notion of equity is the concept of income and risk cross-subsidization [78], whereby the rich cross-subsidize the poor, whilst the healthy cross-subsidize the sick [61]. Notwithstanding this, other scholars have warned that universal population coverage, although desirable, must be carefully pursued to avoid creating a situation of which official entitlements will be offered to all people yet the existing health system may not have sufficient capacity to deliver quality health care for all the population [79, 80]. This is referred to as adverse incorporation or inclusion [79].

### Financial protection as a dimension of universal health coverage

From the perspective of health economics, UHC is viewed as a means of protection against the economic consequences of ill health [81, 82]. A guaranteed financial protection requires the implementation of a health care financing mechanism that does not require direct (substantial) out-of-pocket (OOP) payments, official or informal, such as user fees, copayments and deductibles, for health care at the point of use [23, 74, 81, 83]. This is the reason why the international community has endorsed financing health care from pooled prepaid mechanisms such as tax (general or dedicated) revenue, and contributions from social health insurance (usually for formal sector employees), private health insurance, and micro health insurance as essential pre-requisites for moving towards universal financial protection [6]. The existing literature does not reveal a consensus on the best prepayment mechanism or the right mix of prepayment systems that will guarantee adequate financial protection [22, 84]. A report by Oxfam [22] suggests that within the context of LMICs, different development partners each promote their ideologically favored prepayment mechanisms as a strategy towards achieving UHC. Both the WHO and the academic community, however, recommend that such ideological prescriptions should be abandoned in favor of mixed pooling systems that can coordinate funds from different prepaid sources, in a manner that reflects context-specific UHC needs [2, 28]. This recommendation is also rooted in the recognition that no country, not even high income ones, has achieved complete coverage, relying solely on one single financing strategy [4]. Within a mixed pooling system, there is the need to ensure proper monitory of both private and public inputs that go into the financing system.

The WHO recommends two measures for assessing progress towards financial protection: the incidence of catastrophic health care expenditure and the incidence of impoverishment resulting from OOP payments for health care [25]. The proportion of total health care expenditure incurred through OOP payments is normally used as an indicator of financial protection at the national level [2]. WHO recommends a maximum OOP expenditure threshold of 15–20 % of total health care expenditure as a requirement for financial protection [2]. At the household level, a quantitative measure of financial protection is the proportion of households incurring OOP healthcare expenditure exceeding 40 % of their household’s non-subsistence (i.e., non-food) expenditure [85] or 10 % of total household expenditure [86]. It must be noted that direct medical cost of seeking health care is not the only barrier to financial protection. A good estimate of catastrophic health care expenditure must therefore reflect all relevant costs including non-medical costs such as the cost of travelling to a health facility and loss of earnings while being treated among others. These quantitative measures, estimated on the basis of actual health care cost incurred, however, only reflect the true situation of financial risk protection if all those who need care can actually utilize health services [87]. It is argued that, such utilization-focused quantitative cost estimates are often not able to capture the quantum of needed healthcare that is forgone due to fear of impoverishment associated with utilization [87]. Effective universal financial protection can, therefore, be attained not only if the population does not incur (substantial) OOP payments and critical income losses due to payment for health care, but if there are no fears of and delays in seeking healthcare due to financial reasons, no borrowing and sale of valuable assets to pay for healthcare, and no detentions in hospitals for non-payment of bills [2, 61, 80, 86, 88–90].

### Access to services as a dimension of universal health coverage

From the perspective of public health, it is argued that a UHC package should include a comprehensive spectrum of health services in line with the WHO’s conceptualization of UHC as “*access to key promotive, preventive, curative and rehabilitative health interventions …*” [2, 6]. From a feasibility view point, other scholars, however, argue that the focus should be on the provision of a minimum basic package to cover priority health needs for which there are effective low-cost interventions [91]*.* Some of these scholars insist that this package should include priority services in line with the health-related Millennium Development Goals (MDGs) [14, 24], thereby suggesting a continuous focus on vertical disease-specific interventions. While some of these scholars argue that the expansion and effective implementation of disease-specific interventions, especially those focused on prevention, can improve health and reduce health system costs, opponents insist that all disease-specific interventions create fragmentation and undermine broader efforts aimed at system-wide strengthening [92, 93]. The opposing scholars call for a focus on primary health care [7, 15, 94], to the extent that Yates [74] calls for a clear timetable, proposing 2015 as deadline, for the achievement of universal access to primary health care.

A number of authors further distinguish between official health service coverage, defined in terms of entitlement to services, and actual effective coverage, defined in terms of real access and utilization of health services according to need [13, 44, 51]. It follows that attempts to measure UHC should focus on indicators that measure actual effective service coverage in relation to people’s ability to obtain real access to services, without facing barriers on both the demand and the supply side [13, 51, 70]. Real access, is further defined as access in relation to the availability of health services, personnel and facilities; geographical accessibility of health services; acceptability defined in relation to appropriate client-provider interactions, timeliness, appropriateness and quality of services; and affordability in terms of medical and transport costs of services relative to clients’ ability-to-pay [73, 80, 95–106]. A guaranteed sufficient capacity of the local health system, in terms of adequate health infrastructure, qualified human resources, equipment and tools, to deliver quality health care is therefore an essential component of the access dimension of UHC [2, 11, 107]. It is interesting to note that “*Availability, Acceptability, Affordability and Quality (AAAQ)”* of health services as essential sub-components of real access are directly rooted in the human rights conceptual framework and captured in broader discussions on the right to health [45, 63].

## Summary

Considering its interactive facets, it can be concluded that UHC emerges from the literature as a multi-dimensional concept, operationalized in terms of population coverage of health-related social security systems, financial protection, and access to quality health care according to need [17], and pursued within the framework of health care as an international legal obligation grounded in international human rights laws [45, 46, 48, 49]. As an essential pre-condition for moving towards UHC in LMICs, there is therefore the need for all states to abide by the international human rights obligation imposed on them and thereby legally recognize the right to health in their national constitutions. It is only on this basis that the needed national and political commitment can be enhanced for a successful move towards universal population coverage of health-related social security systems, financial protection and access to services, which are essential components of a guaranteed comprehensive right to health and hence UHC. UHC can thus be understood as a broad legal, rights-based, social humanitarian, health economics and public health concept [15, 17, 27, 42]. As such, it transcends a mere legal extension of the coverage of prepaid financing systems such as health insurance or tax-based systems to all residents, to ensuring that other financial and health system bottlenecks are removed to enhance effective financial protection and equitable access to services for all. As an overall health system strengthening tool, UHC can only be achieved through a human rights-focused integrated approach that recognizes the health system as a complex phenomenon with interlinked functional units whose effective interaractions are essential to reach the equilibrium called UHC. It follows that in LMICs, interventions aimed at strengthening health systems need to attract as much attention and funding as currently being deployed towards disease-specific interventions within the framework of the MDGs. Such an action has the capability of improving local service delivery capacity and hence of building resilient and responsive health systems to facilitate the move towards UHC. The move towards UHC should therefore be conceptualized as a continuous process of identifying gaps in the various interactive UHC dimensions, and designing context-specific strategies to address these gaps in accordance with the international legal obligations imposed on states by international agreements on the right to health. As a global issue, international assistance based on the principle of global solidarity is indispensable in the move towards UHC in LMICs.

## **Abbreviations**

AAAQ: Availability, Acceptability, Affordability, Quality
LMICs: Low - and Middle-Income Countries
MDGs: Millennium Development Goals
OOP: Out-of-pocket
UHC: Universal Health coverage
UNICEF: United Nations Children’s Fund
USAID: United States Agency for International Development
WHA: World Health Assembly
WHO: World Health Organization
